# BIO3 Protocol: Clinical and In Vitro Evidence of Triple Biostimulation With CaHA and P (LA/CL) Threads

**DOI:** 10.1111/jocd.70803

**Published:** 2026-03-25

**Authors:** Luiz Tonon, Maria Oneide Alves, Renata Viana

**Affiliations:** ^1^ Private Practice São Paulo Brazil; ^2^ Ilikia Brasil São Paulo Brazil

## Abstract

**Background:**

Facial aging involves progressive deterioration of the extracellular matrix (ECM), volume loss, and structural sagging. Minimally invasive strategies like absorbable threads provide mechanical support and biostimulation, but their efficacy may be compromised in patients with poor dermal quality. Calcium Hydroxyapatite (CaHA) is a well‐documented biostimulator that enhances collagen and elastin synthesis. This study explored whether combining CaHA with P (LA/CL)‐based absorbable threads could synergistically improve ECM remodeling and clinical outcomes.

**Aims:**

To evaluate the biological and clinical effects of the BIO3 protocol—sequential biostimulation with CaHA and P (LA/CL) threads—by integrating in vitro gene expression analysis and retrospectively collected clinical outcomes.

**Patients/Methods:**

An in vitro model of full‐thickness human skin was used to assess *COL1A1*, *COL3A1*, and *ELN* expression after treatment with CaHA, P (LA/CL) threads, or both. A retrospective clinical study was also conducted with 11 female patients aged 35–65 undergoing facial rejuvenation with threads alone (*n* = 6) or preceded by CaHA (*n* = 5). Clinical outcomes were assessed using GAIS, WSRS, facial laxity scores, ultrasound imaging, and satisfaction questionnaires at multiple timepoints.

**Results:**

Combined in vitro treatment resulted in significantly higher upregulation of ECM‐related genes compared to monotherapies. Clinically, all patients showed aesthetic improvement. The CaHA/P (LA/CL) group demonstrated greater dermal and subcutaneous thickening and higher GAIS ratings at D120. Patient satisfaction reached 100% in both groups by D30 and remained stable. No adverse events were reported.

**Conclusions:**

The BIO3 protocol—combining CaHA and P (LA/CL) threads—showed promising synergistic effects on ECM remodeling and aesthetic outcomes. These findings support the rationale for layered and sequential biostimulation strategies in clinical practice.

## 1 Introduction

Facial aging is a multifactorial process involving volume loss, skin laxity, and diminished collagen production [1]. While thread lifting with absorbable sutures has become a popular minimally invasive technique for facial rejuvenation [2], optimizing the structural integrity of the treated skin remains a challenge. Calcium hydroxyapatite (CaHA) is a well‐documented bio‐stimulator that promotes neocollagenesis, making it a suitable candidate for pre‐treatment prior to mechanical lifting procedures [3].

Previous studies and an international consensus have reported the efficacy of CaHA in restoring facial volume and improving skin quality in Latin American and European populations [4, 5]. APTOS thread lifting is a minimally invasive technique that provides mechanical support and collagen stimulation for facial rejuvenation [6]. It is hypothesized that, in patients with reduced dermal density, diminished adipose support, and compromised structural integrity, the efficacy of thread lifting may be compromised, potentially affecting both the anchorage and longevity of the procedure. Given its well‐documented biostimulatory properties, Calcium Hydroxyapatite (CaHA) emerges as a potential strategy to enhance dermal and subcutaneous tissue quality, thereby improving thread support and integration and overall treatment outcomes.

This study investigates whether the combined action of three collagen biostimulators—CaHA and the bioactive components P (LA/CL) of the APTOS threads—may enhance clinical outcomes.

## 2 Material and Methods

### 2.1 Products

Two commercially available aesthetic medical devices, widely used in minimally invasive facial rejuvenation protocols, were evaluated in this study.

Stiim (by Ilikia, CGbio, Korea) is a sterile, injectable implant composed of 30% calcium hydroxyapatite (CaHA) microspheres suspended in a 70% carboxymethylcellulose (CMC) gel, supplied in a 1.5 mL pre‐filled syringe. It is intended for deep dermal or subcutaneous application. CaHA promotes fibroblast activation and stimulates collagen and elastin synthesis, enhancing skin firmness, elasticity, and long‐term remodeling. For biostimulatory purposes, CaHA is typically diluted prior to injection.

APTOS Visage Excellence (APTOS LLC, Georgia) is an absorbable monofilament thread made of poly (L‐lactide‐co‐ε‐caprolactone) [P (LA/CL)], designed to reposition soft tissues and stimulate neocollagenesis through mechanical tension and local inflammatory signaling.

### 2.2 In Vitro Analysis

#### 2.2.1 In Vitro Skin Model

A full‐thickness human skin equivalent (NV Skin, Nucleo Vitro Laboratory, Brazil) was used to reproduce the structural and functional characteristics of native human skin. The model comprises primary human fibroblasts and macrophages embedded in a protein‐rich dermal matrix, overlaid with keratinocytes to form a stratified epidermis.

The dermal matrix was constructed by incorporating type I collagen into 5× DMEM. The pH was adjusted to neutrality using 0.1 M NaOH, and fibroblasts and macrophages were homogeneously distributed within the collagen scaffold. Keratinocytes were then seeded on top of the matrix. Once confluence was achieved, the construct was lifted to the air–liquid interface to promote epidermal differentiation.

This model enables the evaluation of extracellular matrix production and cellular responses in a physiologically relevant context, as it reproduces the two main layers of the skin—dermis and epidermis—with functional interaction between cell types.

**FIGURE 1:**
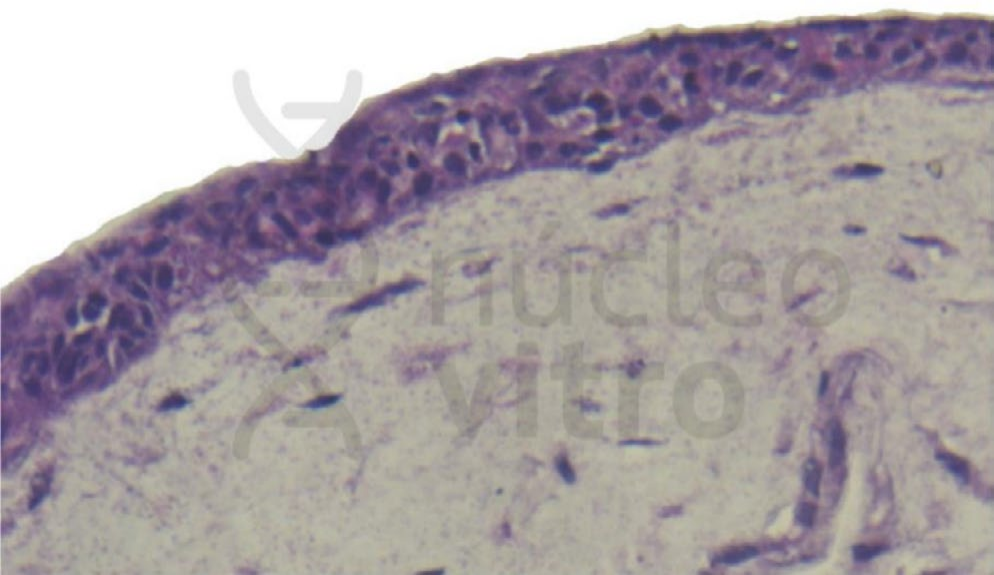
Representative microscopic image of the full‐thickness human skin equivalent (NV Skin) used in this study, stained with hematoxylin and eosin (H&E). *Image kindly provided by Nucleo Vitro Laboratory*.

Primary cells were obtained from the Rio de Janeiro Cell Bank (BCRJ) and maintained under sterile conditions using standard culture media. A representative image is shown in Figure 1.

#### 2.2.2 Experimental Design

To evaluate the individual and combined effects of CaHA and absorbable threads on extracellular matrix remodeling, four experimental conditions were established using the human skin equivalent model: (I) untreated control, (II) CaHA at 1 mg/mL, (III) fragmented P (LA/CL) threads, and (IV) CaHA combined with fragmented P (LA/CL) threads.

For the experimental conditions involving threads (conditions III‐IV), sterile P (LA/CL) sutures were cut under aseptic conditions with sterile scissors into ~4 mm segments. Immediately after cutting, fragments were gently dispersed within the collagen–cell mixture during dermal matrix casting, ensuring that they remained interspersed throughout the dermal compartment after gelation. No additional fragments were introduced into the culture medium at later stages.

Constructs were cultured for 14 days at 37°C in a humidified 5% CO_2_ incubator. At the end of the incubation, samples were collected for gene expression analysis of Extracellular matrix (ECM)‐related markers.

#### 2.2.3 Gene Expression Analysis

To investigate the molecular effects on extracellular matrix remodeling, gene expression analysis was performed via quantitative reverse transcription PCR (RT‐qPCR). After 14 days of culture, total RNA was extracted using Trizol, and purity was confirmed by A260/A280 ratios between 1.8 and 2.0.

Complementary DNA was synthesized from 2000 ng of RNA using the High‐Capacity cDNA Reverse Transcription Kit. RT‐qPCR was performed with SYBR Green and primers for *COL1A1*, *COL3A1*, and *ELN*, using *β‐actin* as the reference gene. Reactions were run in triplicate, and relative expression was calculated using the 2^‐ΔΔCt method, with the untreated control as the calibrator.

#### 2.2.4 Statistical Analysis

All experiments were conducted in triplicate, and data are presented as mean ± standard deviation (SD). Statistical analysis was performed using GraphPad Prism software (version 5). One‐way analysis of variance (ANOVA) followed by Bonferroni's post hoc test was used to assess differences between treatment groups. A *p* < 0.05 was considered statistically significant.

### 2.3 Clinical Study Design

This retrospective observational study included 11 patients who underwent facial rejuvenation with absorbable threads, with or without prior CaHA treatment. All patients were treated in routine aesthetic practice in Brazil. Data were collected from medical records and standardized follow‐up assessments, including patient satisfaction and imaging assessments. Adverse events and complications were documented from chart review.

Inclusion criteria for record selection were females aged 35–65 years treated in the lower and middle thirds of the face. Exclusion criteria included prior facial surgery, severe laxity, or unrelated biostimulatory treatments within the past two years, active dermatologic conditions, or use of medications affecting collagen metabolism. All patients provided informed consent for the use of anonymized data for research purposes.

**FIGURE 2:**
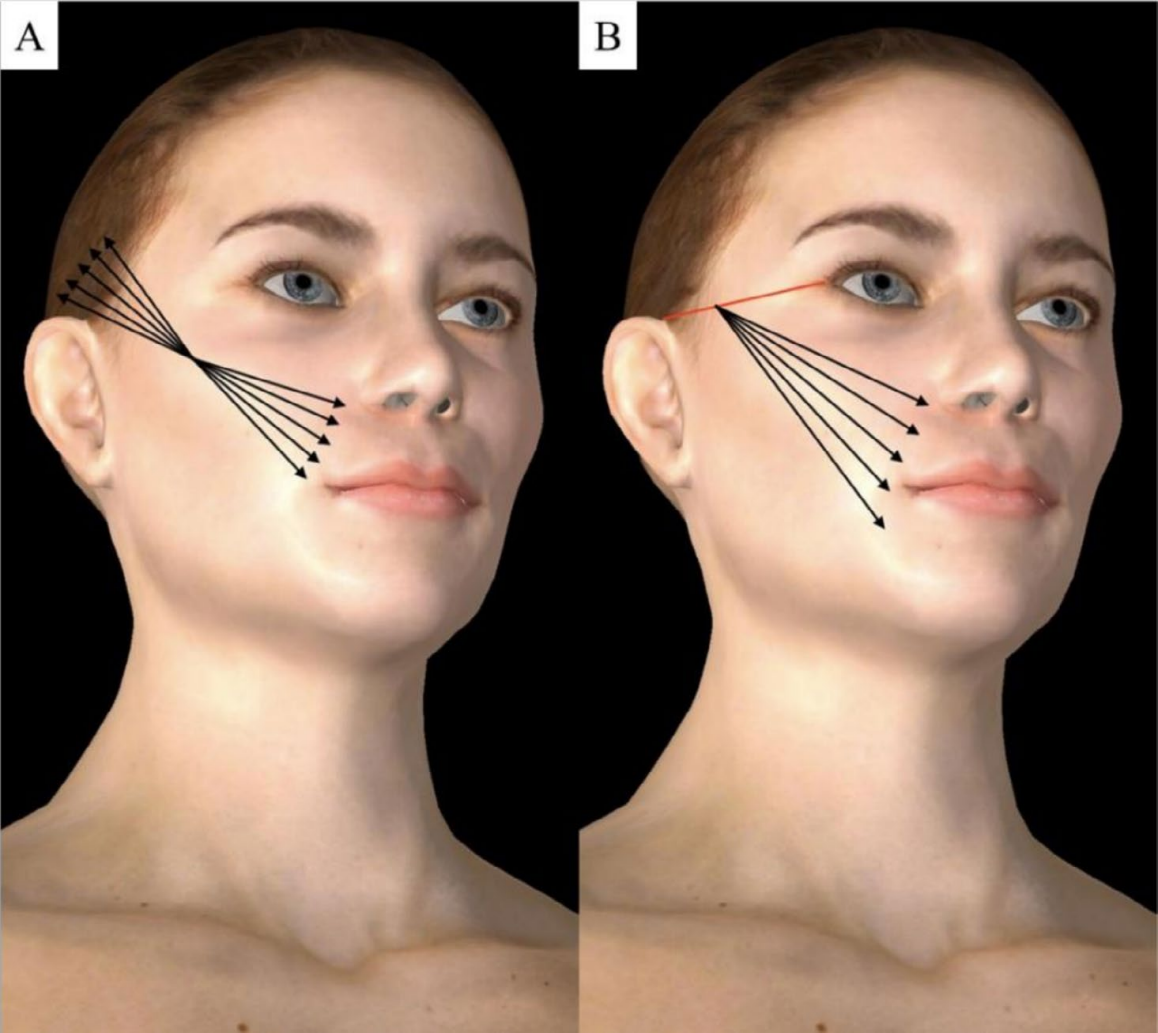
Illustration of the procedure standardized for inclusion in the study: (A) Subdermal injection of calcium hydroxyapatite (CaHA) at a 1:2 dilution ratio using a 22G × 70 mm cannula; (B) Subcutaneous placement of absorbable threads P (LA/CL) using a linear technique.

Only patients in whom the procedure was performed according to the technique illustrated in Figure 2 were included in the analysis, ensuring standardization of both CaHA application and thread placement.

#### 2.3.1 Ethical Considerations

This study adheres to the ethical guidelines of Resolution 466/12 of the National Health Council (CNS) and was approved by the institutional ethics committee under approval number 7.525.844. Patient privacy was safeguarded, and data were used exclusively for scientific purposes.

#### 2.3.2 Statistical Analysis

Paired *t*‐tests were applied to evaluate changes in clinical scales (GAIS, WSRS, and facial laxity). Ultrasound measurements were first tested for normality using the Shapiro–Wilk test. Due to the small sample size and non‐normal distribution in some variables, the Wilcoxon signed‐rank test was used for within‐group comparisons and the Mann–Whitney U test for between‐group comparisons. A *p*‐value < 0.05 was considered statistically significant.

## 3 Results

The results from both the in vitro and retrospective clinical analyses are presented below. The timing adopted between CaHA and thread placement (approximately 60 days) is the routine adopted by the clinic and might reflect the period in which the initial inflammatory response to CaHA has resolved and early neocollagenesis provides improved dermal support.

### 3.1 In Vitro Study

**FIGURE 3:**
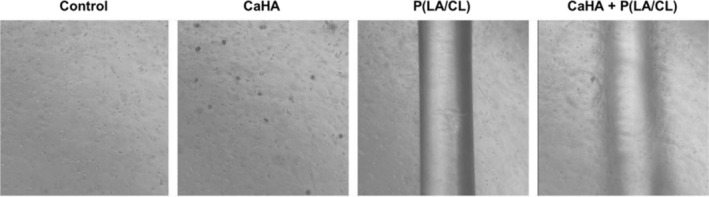
Microscopy of the skin model under different treatment conditions.

After 14 days of exposure to the different treatment conditions, gene expression of *COL1A1*, *COL3A1*, and *ELN* was evaluated by RT‐qPCR to assess extracellular matrix remodeling. Representative microscopic images of the skin equivalents are shown in Figure 3.

Gene expression analysis showed that all treated groups exhibited upregulation of extracellular matrix‐related genes compared to the control.

**FIGURE 4:**
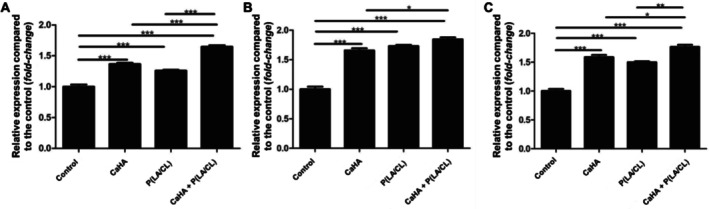
Relative gene expression of *COL1A1*, *COL3A1*, and *ELN* in in vitro skin models after 14 days of treatment. Bars represent fold‐change compared to the control group for the CaHA, P (LA/CL) threads, and combined treatment (CaHA + *P* (LA/CL)) groups. Significant differences between groups are indicated (*p < 0.05, **p < 0.01, ***p < 0.001). Data are shown as mean ± standard deviation.

For *COL1A1* (Figure 4A), expression increased by 36.5% (±3.7) in the biostimulator group, 25.9% (±2.1) in the threads group, and 64.8% (±4.2) in the combined treatment group. Significant differences were observed between the control and all treated groups, as well as between the individual treatments and the combined strategy.

Similar trends were observed for *COL3A1* expression (Figure 4B), which rose by 65.9% (±6.2), 73.1% (±3.4), and 84.5% (±5.7) in the biostimulator, threads, and combined groups, respectively. Once again, the combined group showed the most pronounced effect.

For *ELN* (Figure 4C), the biostimulator and threads groups exhibited increases of 58.8% (±6.3) and 49.9% (±2.7), respectively, while the combination of both led to a 76.6% (±5.9) increase. Statistical comparisons confirmed significant improvements in all treated groups compared to the control, with the combined treatment group outperforming the individual treatments.

### 3.2 Clinical Data

Participants included in the retrospective analysis were classified into two groups according to the treatment received: those who underwent CaHA injection followed by thread placement (CaHA/P (LA/CL)), and those who received thread treatment alone (P (LA/CL)). In the CaHA/P (LA/CL), data were collected from clinical records corresponding to D‐60 (baseline, before CaHA), D0 (60 days post‐CaHA, before threads), D30, and D120 (30 and 120 days after thread placement, respectively). In the threads group, evaluations were conducted at D0 (baseline), D30, and D120, relative to thread placement.

#### 3.2.1 Study Population and Baseline Characteristics

A total of eleven female patients were included in the clinical analysis. Among them, six underwent facial rejuvenation with absorbable threads alone (P (LA/CL)), while five received calcium hydroxyapatite (CaHA) 60 days prior to thread implantation (CaHA/P (LA/CL)).

Baseline characteristics were comparable between groups. The mean age was 45.6 years (SD 2.9) in the CaHA/P (LA/CL) group and 44.5 years (SD 6.5) in the P (LA/CL) group. Body mass index (BMI) averaged 25.6 (SD 1.2) and 27.0 (SD 3.3), respectively. Two patients in the CaHA/P (LA/CL) group reported tobacco use, while none did in the other group. These data are summarized in Table 1.

**TABLE 1:** Baseline demographic and clinical characteristics of study patients undergoing facial rejuvenation with absorbable threads alone or in combination with prior biostimulator treatment (CaHA).

Baseline characteristics of participants		
Group	CaHA/P (LA/CL)	P (LA/CL)
Participants	5	6
Age (mean, SD)	45.6, 2.9	44.5, 6.5
BMI in kg/m2 (mean, SD)	25.6, 1.2	27, 3.3
Tabagism	2	0

Regarding prior aesthetic procedures, four patients in the CaHA/P (LA/CL) group and one patient in the P (LA/CL) group had received botulinum toxin at least 4 months before inclusion, and one patient in the P (LA/CL) group had a history of prior laser and microfocused ultrasound treatment.

#### 3.2.2 Clinical Evaluation

**FIGURE 5:**
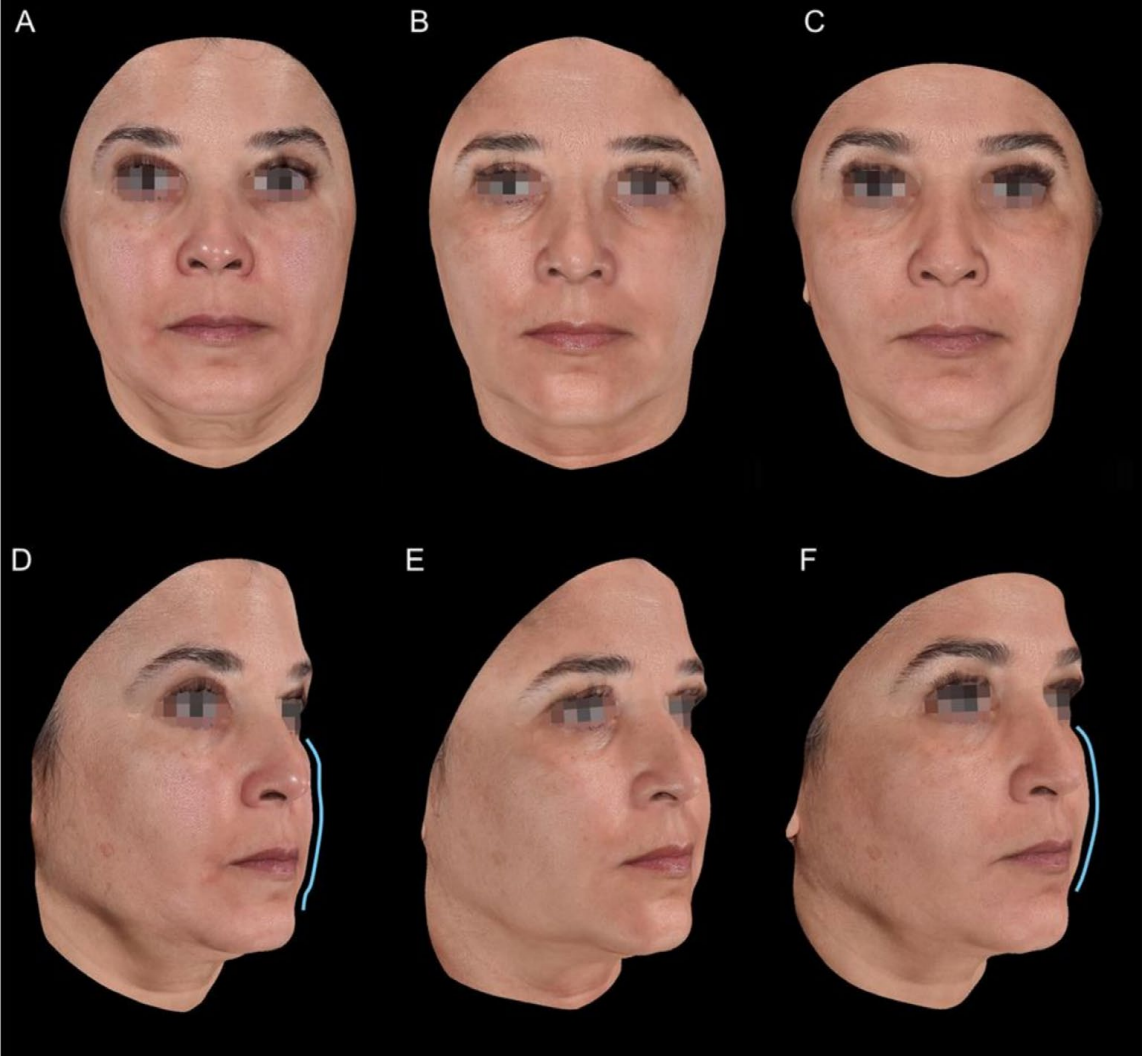
Representative 3D photographic images of a patient from the CaHA/P (LA/CL) group at baseline (A, D), 30 days after thread placement (B, E), and 120 days after thread placement (C, F). Repositioning of fat compartments in the mid and lower face is highlighted in blue.

**FIGURE 6:**
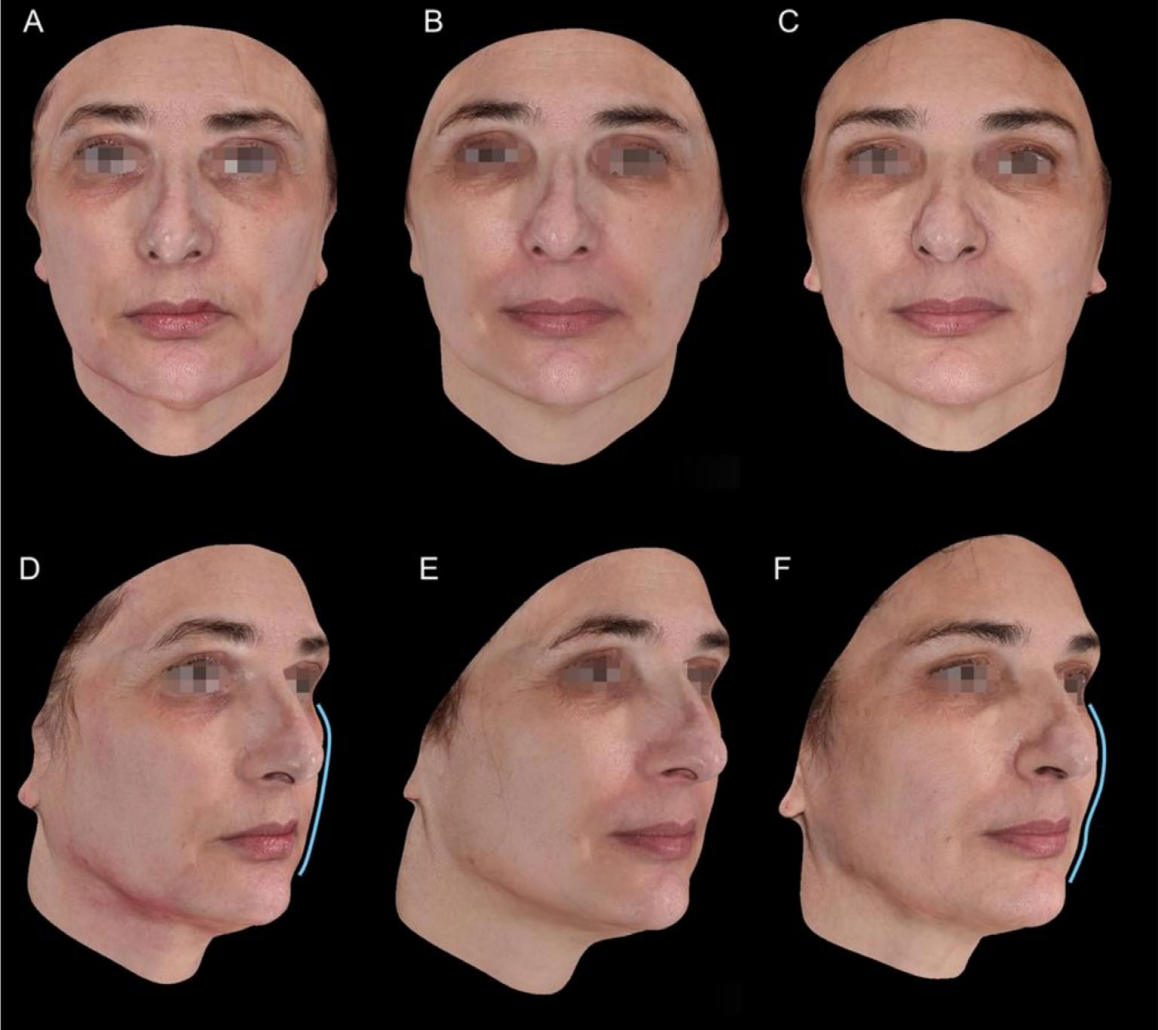
Representative 3D photographic images of a patient from the P (LA/CL) group at baseline (A, D), 30 days after thread placement (B, E), and 120 days after thread placement (C, F). Repositioning of fat compartments in the mid and lower face is highlighted in blue.

Photographic assessments, including both 2D and 3D imaging, from follow‐up visits were reviewed to document aesthetic improvements. Representative images illustrating changes in wrinkle depth, skin firmness, and overall contour enhancement are presented in Figures 5 and 6. Repositioning of facial fat compartments was also documented, particularly in the mid and lower face, contributing to improved definition and support. These changes are highlighted in blue in Figures 5 and 6. Clinical evaluation was based on the Global Aesthetic Improvement Scale (GAIS), the Wrinkle Severity Rating Scale (WSRS), and a facial laxity score, based on standardized photographs assessed by three independent physicians. Mean values were calculated for all scales.

##### 3.2.2.1 GAIS Assessment

At D30, all participants in both groups were rated as improved or better on the GAIS. In the CaHA/P (LA/CL) group (*n* = 5), ratings at D30 were 60% (3 patients) “very much improved,” 20% (1 patient) “much improved,” and 20% (1 patient) “improved.” In the threads‐only group (*n* = 6), D30 ratings were 33% (2 patients) “very much improved,” 17% (1 patient) “much improved,” and 50% (3 patients) “improved.” This clinical improvement was maintained at D120. At that time point, the CaHA/P (LA/CL) group showed 40% (2 patients) “very much improved,” 40% (2 patients) “much improved,” and 20% (1 patient) “improved,” while the threads‐only group showed 83.3% (5 patients) “much improved” and 16.7% (1 patient) “improved.” No participant was classified as “no change” or “worse” at any assessment. Given the small sample size and the ordinal nature of the GAIS, results were reported descriptively without inferential statistics.

##### 3.2.2.2 WSRS Assessment

In the P (LA/CL) group, mean wrinkle severity scores improved from 2.4 at baseline (D0) to 2.0 at D30 and 1.9 at D120. In the CaHA/P (LA/CL) group, scores also showed improvement from 2.7 at baseline (D0) to 2.5 at D30 and 2.3 at D120. Wrinkle severity significantly decreased from baseline to D120 when considering all patients (*p* = 0.003). No significant between‐group differences were observed at D30 (*p* = 0.205) or D120 (*p* = 0.398).

##### 3.2.2.3 Laxity Assessment

In the threads P (LA/CL) group, the mean facial laxity scores improved from 3.2 at D0 to 2.4 at D30 and 2.2 at D120. In the CaHA/P (LA/CL), scores were 3.2 at D0, 2.7 at D30, and 2.9 at D120. A significant reduction from baseline to D120 was observed across all patients (*p* = 0.003). Between‐group differences were not significant at D30 (*p* = 0.285) but became significant at D120 (*p* = 0.027), favoring the threads group.

#### 3.2.3 Patient Satisfaction

**FIGURE 7:**
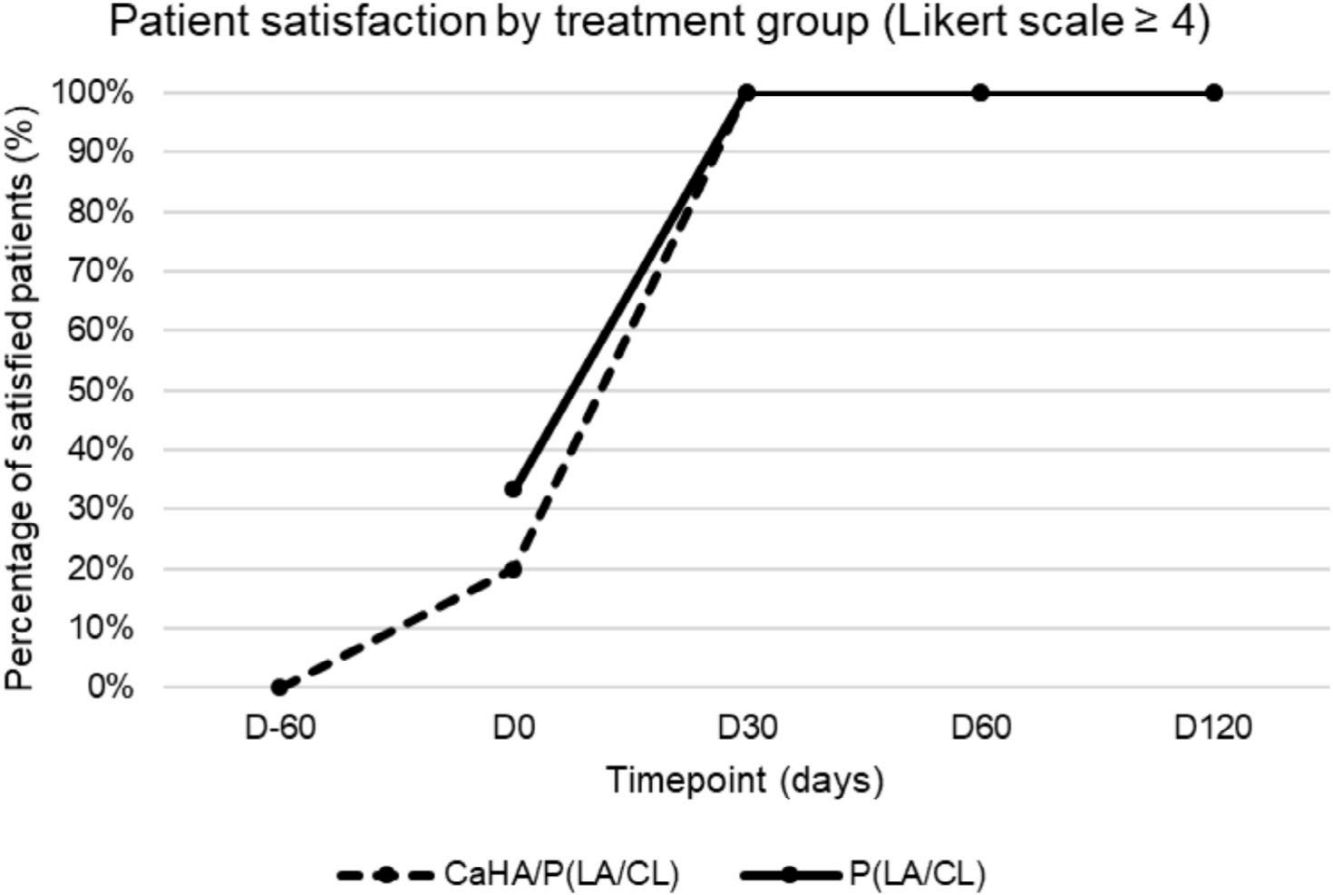
Percentage of satisfied patients (Likert score ≥ 4) at each timepoint in both treatment groups. D0 is considered the day of thread implantation.

Self‐reported satisfaction data were obtained from medical records using a five‐point Likert scale, with scores ≥ 4 considered positive (Figure 7). Before any treatment (D–60), no patients in the CaHA/P (LA/CL) group reported satisfaction. By D0 (day of thread implantation), 20% of CaHA/P (LA/CL) patients and 33% of those in the P (LA/CL) threads group reported satisfaction, indicating a possible effect of prior biostimulator application. At D30, satisfaction reached 100% in both groups and remained stable through D60 and D120.

At 60 days after CaHA application, just before thread implantation (D0), 80% of patients in the CaHA/P (LA/CL) group were already classified as “improved” or “much improved”, according to available photographic documentation, indicating a visible response to CaHA. At D30 post‐thread implantation, all patients in both groups showed improvement. In both the CaHA/P (LA/CL) and P (LA/CL) groups, 60% were rated as “very much improved,” with the remaining patients evenly distributed between “much improved” and “improved.” By D120, 100% of patients maintained some level of improvement. However, the highest GAIS category (“very much improved”) was observed only in the CaHA/P (LA/CL) group (40%), while patients in the threads group were rated as either “much improved” or “improved.”

No cases of “no change” or “worsening” were reported at any timepoint.

#### 3.2.4 Ultrasound Analysis

Ultrasound data obtained from follow‐up examinations revealed structural changes in all skin layers, particularly in the CaHA/P (LA/CL) group. Between D–60 (CaHA injection) and D0 (thread implantation), this group showed a 45.6% reduction in epidermal thickness, along with increases of 9.6% in dermal thickness and 25.4% in subcutaneous thickness.

Thirty days after thread implantation (D30), both groups exhibited changes, with greater magnitude in the CaHA/P (LA/CL). Epidermal thickness decreased by 42.1% in this group versus −2.6% in the P (LA/CL) threads group (*p* = 0.06). Dermal and subcutaneous thickness increased by 9.2% and 18.5%, respectively, in the CaHA/P (LA/CL) group, compared to 4.2% and 8.7% in the threads group (*p* > 0.05 for all comparisons).

At D120, the CaHA/P (LA/CL) group showed a−36.8% reduction in epidermal thickness, with increases of 20.4% in dermal thickness and 14.3% in subcutaneous tissue. In contrast, the threads group showed no change in epidermal thickness (0.0%), and modest increases in the dermis (2.6%) and subcutis (11.3%). A trend toward significance was observed for dermal thickness between groups at this timepoint (*p* = 0.082).

**FIGURE 8:**
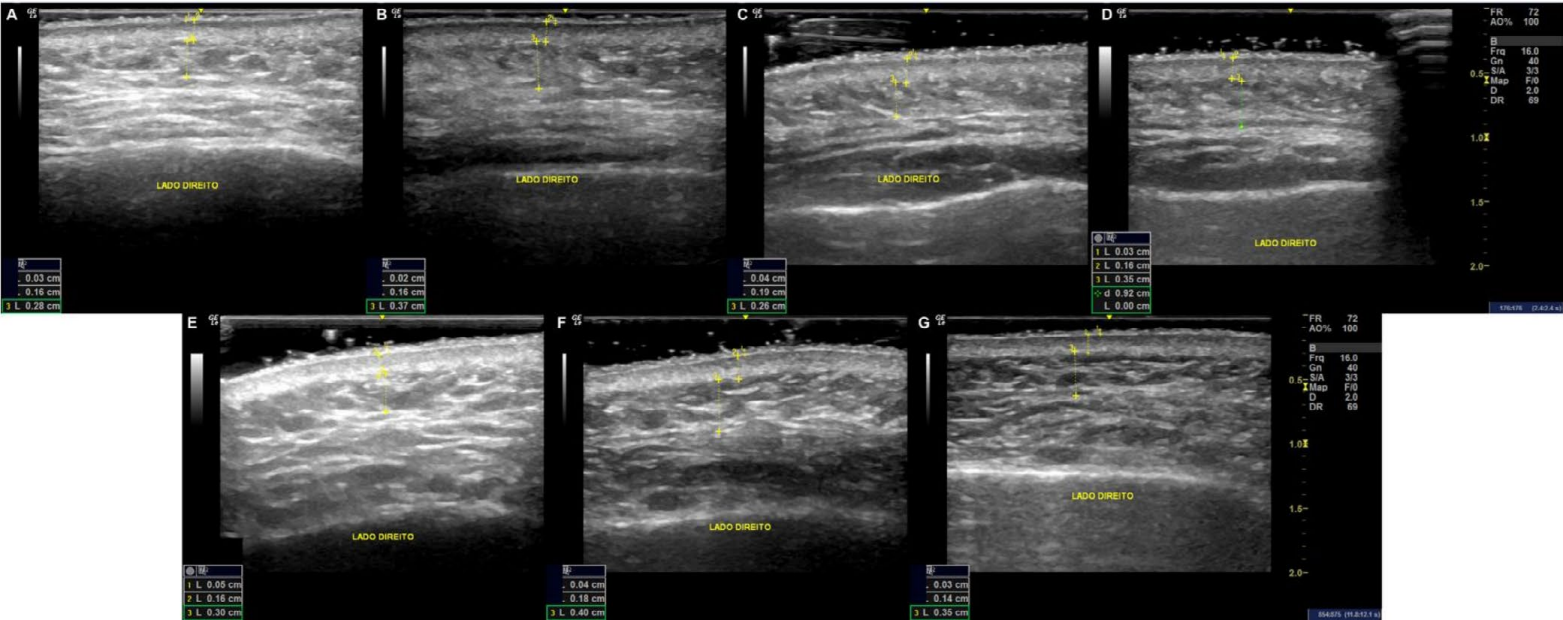
Representative ultrasound images from patients in the CaHA/P (LA/CL) group (A–D) and P (LA/CL) group (E–G), showing structural changes over time. In the CaHA/threads group, images correspond to D–60 (A), D0 (B), D30 (C), and D120 (D). In the threads group, images correspond to D0 (E), D30 (F), and D120 (G).

Percentage variations for all layers and timepoints are summarized in Table 2, and representative ultrasound images are presented in Figure 8.

**TABLE 2:** Percentage variation in epidermal, dermal, and subcutaneous thickness over time, as measured by ultrasound, in the combined treatment group (biostimulator + threads) and threads group. Changes are expressed relative to baseline values.

Skin thickness variation—CaHA/threads			
	D‐60—D0	D‐60—D30	D‐60—D120
Epidermis	−45,6%	−42,1%	−36,8%
Dermis	9,6%	9,2%	20,4%
Subcutaneous	25,4%	18,5%	14,3%

Skin thickness variation—threads		
	D0—D30	D0‐ D120
Epidermis	−2,6%	0,00%
Dermis	4,2%	2,6%
Subcutaneous	8,7%	11,3%

## 4 Discussion

Combined treatment strategies have gained attention for addressing these changes more comprehensively. The Bio3 protocol analyzed in this study incorporates two products containing three established collagen biostimulators ‐ CaHA, PLLA, and PCL‐ potentially combining early fibroblast activation and thread lifting to long‐term tissue remodulation. The rationale of this approach lies in combining biochemical stimulation and mechanical reinforcement to optimize dermal regeneration and facial support.

Each compound in the BIO3 protocol contributes to dermal regeneration through specific mechanisms of action. Calcium hydroxyapatite (CaHA) acts by stimulating fibroblasts and promoting the production of collagen types I and III, elastin, and new blood vessels, while also modulating inflammation and improving tissue structure [7]. Poly‐L‐lactic acid (PLLA) induces a controlled inflammatory response that leads to fibroblast activation via the TGF‐β/Smad signaling pathway, resulting in collagen synthesis and tissue remodeling, with additional effects on adipocyte activity and anti‐inflammatory macrophage polarization [8]. Polycaprolactone (PCL), present in absorbable P (LA/CL) threads, promotes long‐term neocollagenesis by forming a stable three‐dimensional scaffold that interacts with fibroblasts, supporting sustained collagen type I production, elastin formation, and neovascularization [9]. In P (LA/CL) blends, incorporation of PCL improves mechanical stability and slows PLLA degradation, which may prolong tissue support and remodeling [10]. When used sequentially, these agents may act synergistically, enhancing and prolonging the tissue remodeling process [3].

In vitro results demonstrated synergistic upregulation of *COL1A1*, *COL3A1*, and *ELN* expression, exceeding the expression seen with either treatment alone, suggesting that mechanical and biochemical stimuli may potentiate fibroblast activity. These findings support the biological plausibility of combining CaHA and absorbable threads for layered tissue ECM remodeling and sustained dermal stimulation.

In the retrospective clinical analysis, both treatment groups showed aesthetic improvement, but with distinct patterns. The CaHA/P (LA/CL) group exhibited greater dermal and subcutaneous thickening on ultrasound, whereas the threads‐only group demonstrated slightly better laxity scores at D120. These findings suggest that CaHA primarily contributes to dermal densification and extracellular matrix (ECM) remodeling, while mechanical lifting with threads provides more immediate tissue repositioning.

Ultrasound analysis revealed the most notable structural differences between groups. Patients who received CaHA prior to thread placement exhibited greater increases in dermal and subcutaneous thickness, accompanied by persistent hyperechoic signals up to 3 months post‐injection. These findings likely reflect ECM densification resulting from increased collagen and elastin synthesis. The reduction in epidermal thickness observed in some cases may correspond to volumetric expansion of deeper layers, rather than epidermal atrophy. A denser dermal matrix may also enhance thread anchorage and facilitate the repositioning of facial fat compartments, as illustrated by 3D photographic documentation. The observed subcutaneous thickening could further contribute to thread stability by providing a more cohesive and supportive environment. While plausible, these mechanisms remain hypothetical and were not confirmed histologically. No adverse events were reported, supporting safety and tolerability of the BIO3 protocol in this clinical setting.

This analysis has important limitations, including the small sample size, retrospective design, and absence of intraindividual controls, which reduce statistical power and generalizability. Therefore, these findings should be interpreted as preliminary and hypothesis‐generating. Further controlled studies with larger samples and longer follow‐up periods are required to validate these observations. Despite these constraints, it provides a multidimensional understanding of tissue response to sequential biostimulation integrating in vitro findings, ultrasound imaging, and clinical outcomes. These preliminary results support the rationale for layered and timed collagen stimulation and warrant confirmation in larger, controlled prospective studies to validate the clinical relevance of the protocol.

## 5 Conclusion

The retrospective findings from this study indicate that the BIO3 protocol integrates three established collagen biostimulators—CaHA, PLLA, and PCL—applied in a sequential and layered manner. The integration of in vitro, imaging, and clinical data suggests an association with improvements in dermal structure and soft tissue support. Although further prospective studies are needed to confirm these results and elucidate mechanisms, the BIO3 technique appears to be a feasible and structured strategy for facial tissue regeneration in aesthetic practice.

Although the results suggest synergistic effects between CaHA and P (LA/CL) threads, the small retrospective sample limits definitive conclusions. Future prospective studies with larger populations are needed to confirm the observed trends.

## Funding

Luiz Tonon and Maria Oneide provide lectures and training sessions on the products used in this article and also serve as consultants for Ilikia Brasil, the distributor of these products in Brazil. Renata Viana serves as a scientific consultant for Ilikia. The authors declare no potential conflicts of interest with respect to the research, authorship, or publication of this article.

## Conflicts of Interest

The authors declare no conflicts of interest.

## Data Availability

The data that support the findings of this study are available from the corresponding author upon reasonable request.
